# Comparative genetic structure of two mangrove species in Caribbean and Pacific estuaries of Panama

**DOI:** 10.1186/1471-2148-12-205

**Published:** 2012-10-18

**Authors:** Ivania Cerón-Souza, Eldredge Bermingham, William Owen McMillan, Frank Andrew Jones

**Affiliations:** 1 Smithsonian Tropical Research Institute, Apartado, 0843-03092, Panama; 2 University of Puerto Rico, Rio Piedras Campus, PO BOX 23360, San Juan, 00931-3360, Puerto Rico; 3 Department of Botany and Plant Pathology, Oregon State University, 2070 Cordley Hall, Corvallis, OR, 97331, USA

**Keywords:** Rhizophora mangle, Avicennia germinans, Pollen dispersal, Seed dispersal, Spatial genetic structure

## Abstract

**Background:**

Mangroves are ecologically important and highly threatened forest communities. Observational and genetic evidence has confirmed the long distance dispersal capacity of water-dispersed mangrove seeds, but less is known about the relative importance of pollen vs. seed gene flow in connecting populations. We analyzed 980 *Avicennia germinans* for 11 microsatellite loci and 940 *Rhizophora mangle* for six microsatellite loci and subsampled two non-coding cpDNA regions in order to understand population structure, and gene flow within and among four major estuaries on the Caribbean and Pacific coasts of Panama.

**Results:**

Both species showed similar rates of outcrossing (t= 0.7 in *A. germinans* and 0.8 in *R. mangle*) and strong patterns of spatial genetic structure within estuaries, although *A. germinans* had greater genetic structure in nuclear and cpDNA markers (7 demes > 4 demes and *Sp*= 0.02 > 0.002), and much greater cpDNA diversity (*H*_*d*_= 0.8 > 0.2) than *R. mangle*. The Central American Isthmus serves as an exceptionally strong barrier to gene flow, with high levels nuclear (*F*_*ST*_= 0.3-0.5) and plastid (*F*_*ST*_= 0.5-0.8) genetic differentiation observed within each species between coasts and no shared cpDNA haplotypes between species on each coast. Finally, evidence of low ratios of pollen to seed dispersal (r = −0.6 in *A. germinans* and 7.7 in *R. mangle*), coupled with the strong observed structure in nuclear and plastid DNA among most estuaries, suggests low levels of gene flow in these mangrove species.

**Conclusions:**

We conclude that gene dispersal in mangroves is usually limited within estuaries and that coastal geomorphology and rare long distance dispersal events could also influence levels of structure.

## Background

Mangrove communities are composed of phylogenetically unrelated species, each adapted in different ways to the coastal environment where high salinity, diurnal patterns of submergence, wave action, and frequent disturbance create high stress environments [1,2]. Mangrove forests have an extended tropical and subtropical geographic distribution during the last 40 My [3,4]. They are also among the most biologically productive forests and provide key ecosystem services such as breeding grounds for fish, shrimp, and birds and function to protect coasts from tidal surges during hurricanes or extreme weather [5,6]. However, mangrove forests are being destroyed and fragmented at alarmingly high rates in the tropics due to human development [7-10]. Moreover, mangrove forests may be particularly susceptible to increasing sea levels [11]. The ability of mangroves to adapt and regenerate in the face of disturbance and changing environmental conditions is therefore dependent upon rates of local and long distance dispersal, colonization and genetic diversity found within and among populations.

In most trees, it is generally assumed that rates of pollen gene dispersal are greater, and often much greater, than rates of gene flow via seed. However, several lines of evidence suggest that mangrove species are capable of frequent long distance seed dispersal (LDD) on scales of many km that may exceed rates of gene flow via pollen. For example, exceptionally high levels of gene flow have been reported between West Africa and South America at scales > 6,000 km [12,13]. In addition, mark and recapture experiments in ocean currents in the Caribbean and in the Gulf of Mexico have revealed high dispersal potential for mangrove seeds [14-17]. Direct measurements at very local scales within mature mangrove forests have shown very short seed dispersal distances i.e. less than 10 m in two weeks, [18]. Therefore, the extent of gene flow via seed observed among populations may be due to the frequency at which seeds encounter large open ocean currents when dispersed from more isolated estuaries where water flow is largely due to diurnal tidal action.

**Figure 1:**
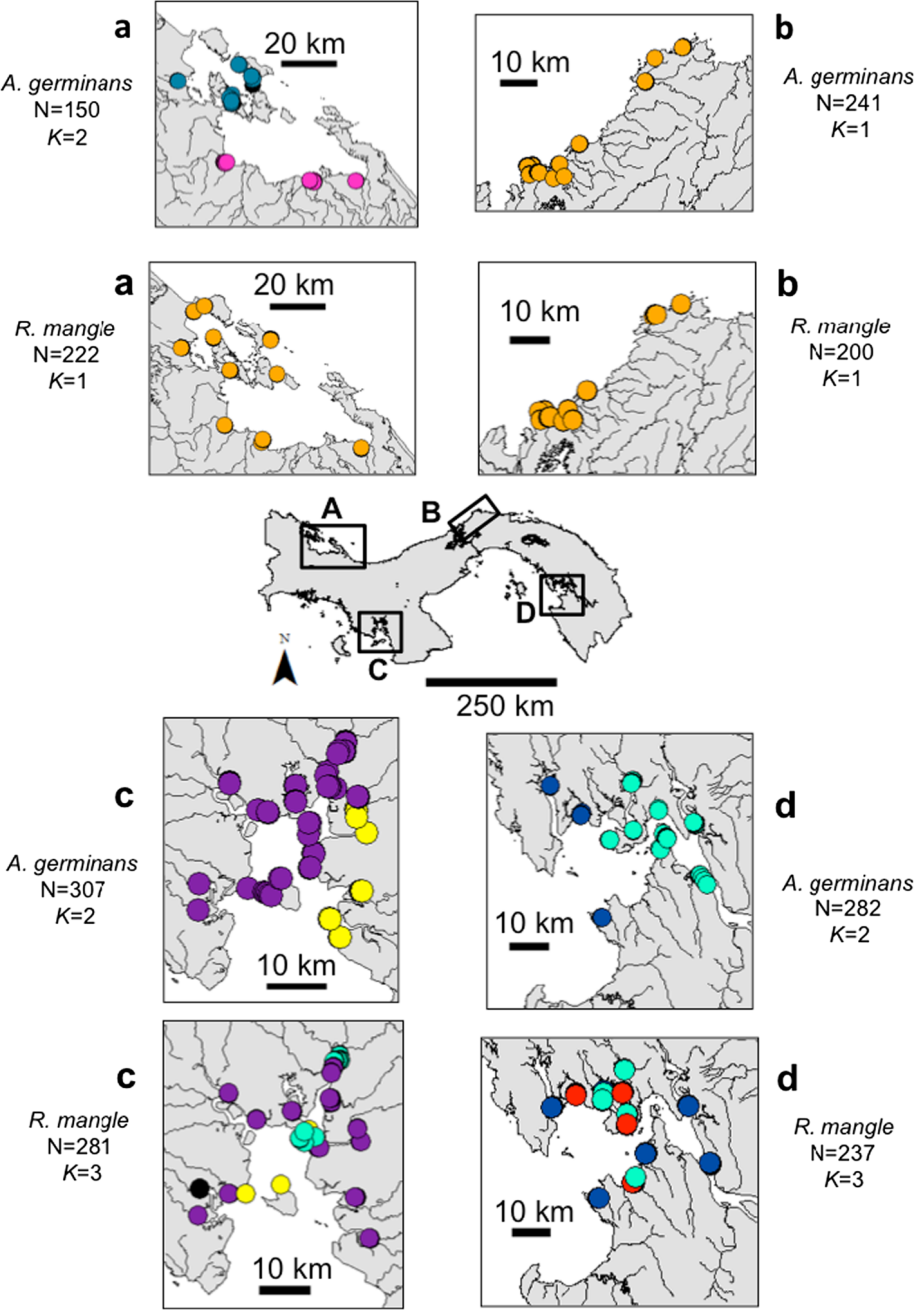
**Population structure for two mangrove species within each estuary when analyzed separately using GENELAND 2.0.12.** The estuaries under study were Bocas del Toro (**a**) and Costa Arriba (**b**) in the Caribbean, and Montijo Gulf (**c**) and San Miguel Gulf (**d**) in the Pacific. Points represent individuals and their colors represent the assignment of each one of the inferred clusters (*K*). Admixed individuals that were simultaneously assigned to two clusters within each estuary with a probability > 0.5 are in black. Due to the scale of estuary maps, all individuals from same transect (i.e. ~30 individuals) are overlapping. However, they were usually assigned to the same cluster (i.e. same color in the map) with very few exceptions.

A comparison between the extent of pollen vs. seed gene flow in mangroves would improve our understanding of mangrove population structure and reveal the contribution of each to observed spatial patterns of genetic structure [19-21]. In this study, we compare patterns of nuclear and plastid genetic structure in the two dominant mangrove species from the new world, the black mangrove (*Avicennia germinans,* Avicenniaceae) and the red mangrove (*Rhizophora mangle,* Rhizophoraceae), across the four main estuaries of Panama within a scale up to 300 km along same coastline (Figure 1). The focal species currently occur in sympatry on both sides of the Central American Isthmus (CAI) but have different historical biogeographies. *Rhizophora mangle* has been present in the Neotropics for 40 My while *A. germinans* has been present since 16 My [3,4,22]. The two species have contrasting patterns of life-history traits and current demography. *Avicennia germinans* has an entomophilous pollination system with polyphile (i.e. bees, wasps and flies identified as effective pollinators), whereas *R. mangle* is characterized by a simultaneous wind (anemophily) and entomophilous pollination, termed ambophilous pollination [2,23-26]. Although the two mangrove species have perfect flowers, the breeding mechanism seems to be different [2]. The protandry observed in *A. germinans* suggest a mostly outcrossing breeding system, however self-compatibility has not been tested yet in this species [24]. In comparison, *R. mangle* shows a mixed-mating breeding system where self-pollination could be more frequent [23,27]. *Avicennia germinans* has small light ovoid crypto-viviparous propagules with longevity of four months while *R. mangle* seeds are large, elongate and viviparous with longevity of one year [2,28,29]. In addition, while *R. mangle* is the only species from the genus *Rhizophora* to inhabit the Caribbean coast of Panama, on the Pacific side it is in sympatry with *R. racemosa,* generating introgressive hybrid zones where morphological and genetic distinctions between species and hybrids are intricate [13]. Finally, populations of *A. germinans* have a patchy, low-density distribution (e.g. 6 stems/km^2^ in Bocas del Toro, Panama) compared to *R. mangle* populations, which have a more uniform distribution with extremely high densities (e.g. 1,544 stems/km^2^ in Bocas del Toro, Panama) [30]. Previous analyses of *A. germinans* have reported high levels of genetic diversity of this species on the Pacific side of CAI, including Panama [12,31,32].

Based on these life-history characteristics and previous reports of seed dispersal capacity, we expected that: i) both species would display low genetic structure or little evidence for isolation by distance (IBD) within estuaries and ii) both species would have a higher rate of seed gene flow than pollen gene flow because of their capacity for LDD via sea-drift seeds [12,13]. In addition, at the species level comparison we expected that *Avicennia germinans* would have greater degree of population differentiation than *R. mangle* because a combination of lower seed longevity and viability in ocean water, insect pollen dispersal mechanism, and low-density distribution [19,33].

## Results

### Genetic differentiation and mating systems

Despite differences in many life history characteristics, the two species did not show significant differences in levels of genetic structure (*F*_*ST*_ = 0.32 ± 0.04 SE for *A. germinans* and *F*_*ST*_ = 0.40 ± 0.05 SE for *R. mangle*), inbreeding (*F*_*IS*_ = 0.20 ± 0.03 SE for *A. germinans* and *F*_*IS*_ = 0.13 ± 0.13 SE for *R. mangle*) or outcrossing rates (*t* = 0.67 ± 0.06 SE for *A. germinans* and *t* = 0.77 ± 0.19 SE for *R. mangle*) (Kruskal-Wallis ANOVA, P >0.05, N=4) (Table 1). Both species showed deviations from HWE, but only *R. mangle* showed evidence of linkage disequilibrium. These deviations could be explained by deme structure within estuaries that we explained below.

**Table 1:** **Nuclear microsatellite *F***_***ST ***_**and Chloroplast (cpDNA) *F***_***ST ***_**estimated as *G***_***ST ***_**in two mangrove species**

**Mangrove species**	**Microsatellite**			**CpDNA**				
	***F***_***ST***_	***F***_***IS***_	**Outcrossing level**	**Hamilton & Miller’s method**				**Ennos ’s method**
	**(SE)**	**(SE)**	**(t) (SE)**	**Expected cpDNA *F***_***ST***_	**95% CI**	**Observed cpDNA *G***_***ST***_	**95% CI**	**r = Pollen flow/Seed flow**
*Avicennia germinans*	0.3181	0.1950	0.6736	0.5550	0.4758-0.6342	0.3469	0.1407-0.5531	−0.64
	(0.0396)	(0.0335)	(0.0597)					
*Rhizophora mangle*	0.4001	0.1327	0.7657	0.6485	0.5461-0.7509	0.8504	0.5888-1.1120	7.65
	(0.0512)	(0.1307)	(0.1885)					

The estimates of *G*_*ST*_ were calculed using the Hamilton and Miller’s method [21, eq. 10] and the Ennos’s method [43, eq. 5a] in *Avicennia germinans* (black mangrove) and *Rhizophora mangle* (red mangrove) across four Panamanian estuaries. The comparison of *F*_*ST*_, *F*_*IS*_ and outcrossing level (t) between *A. germinans* and *R. mangle* did not show significant differences between species (Kruskall-Wallis ANOVA, P>0.05, N=4).

### General patterns of diversity among four estuaries

Both species showed striking differences in genetic diversity. *Avicennia germinans* showed considerable variation in microsatellite alleles among four estuaries, with the highest *H*_*e*_ observed in the Caribbean Costa Arriba estuary (0.730) and lowest in the Pacific San Miguel Gulf (0.459). In contrast, *R. mangle* showed lower overall gene diversity in Caribbean populations (0.349 and 0.305 for Bocas del Toro and Costa Arriba, respectively) relative to Pacific estuaries (0.654 and 0.610 for Montijo Gulf and San Miguel gulf, respectively), however, no significant differences in outcrossing rates within species were observed among estuaries (Table 1 and Additional file 1). The higher gene diversity and number of alleles in Pacific estuaries of *R. mangle* is complicated by the complex hybridization evident in both Pacific estuaries (Figure 1), which serves as a novel source of genetic diversity in Pacific populations [13].

**Figure 2:**
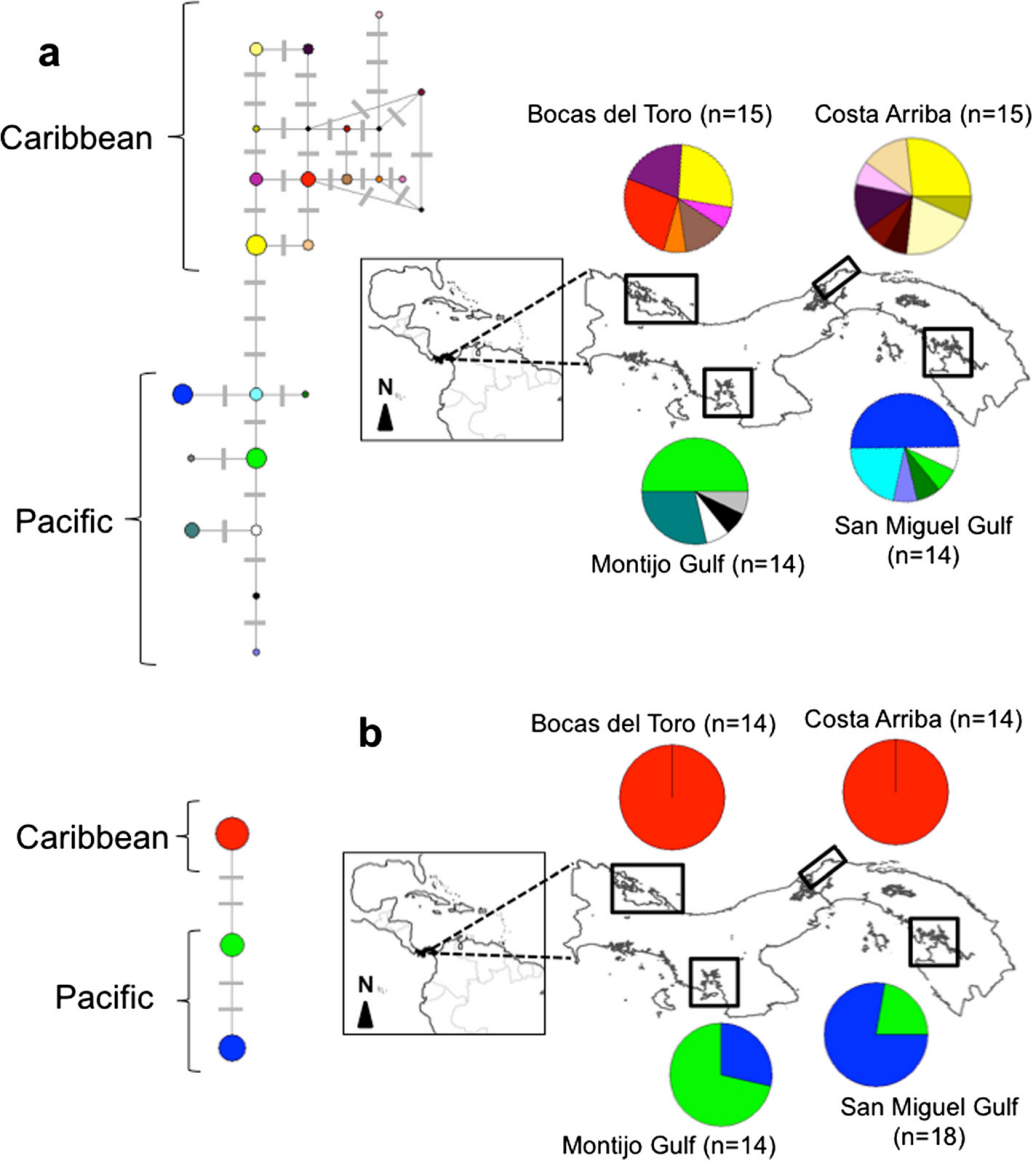
**Median joining networks and geographic distribution of cpDNA haplotypes across four estuaries in Panama.** (**a**) *Avicennia germinans* (Black mangrove) and (**b**) *Rhizophora mangle* (Red mangrove). No haplotypes were shared across the Central American Isthmus.

Chloroplast DNA also revealed striking patterns in diversity and structure in these two species when comparing among estuaries. *Rhizophora mangle* has a single fixed haplotype in the Caribbean coast that include Bocas del Toro and Costa Arriba estuaries (*H*_*d*_ = 0.00), and only two haplotypes in the Pacific (*H*_*d*_ = 0.44 for Montijo Gulf and *H*_*d*_ = 0.37 for San Miguel Gulf) for a total of three haplotypes. The opposite trend was observed in *A. germinans*, which showed remarkably high genetic diversity of cpDNA haplotypes with 22 haplotypes observed from 58 individuals. For this species, the haplotype diversity was similar among four estuaries (*H*_*d*_ = 0.84 for Bocas del Toro, *H*_*d*_*=* 0.89 for Costa Arriba, 0.70 for Montijo Gulf and 0.73 for San Miguel Gulf). Finally, no cpDNA haplotypes were shared between coasts for either species (Figure 2, Additional file 1, Additional file 2 and Additonal File 3).

### Hierarchical structure of genetic variation based on microsatellites

We found evidence of similar levels of genetic structure between species overall (*F*_*ST*_ = 0.32 ± 0.04 SE for *A. germinans F*_*ST*_ = 0.40 ± 0.05 SE for *R. mangle*, Table 1). In addition, both species showed that majority of microsatellite variation is partitioned within estuaries (70% for *A. germinans* and 64% for *R. mangle*) with differences among estuaries accounting for most of the remaining variation (30% and 36%, Table 2). Thus, pairwise comparison of estuaries separated by the ocean showed exceptionally high levels of differentiation with pairwise *F*_*ST*_ ranged between 0.31 to 0.46 in *A. germinans* and between 0.47 to 0.51 in *R. mangle.* Corrected estimates of *F*_*ST*_ by null allele presence were highly similar to non-corrected *F*_*ST*_ values putatively harboring null alleles (Table 3, Additional file 4).

**Table 2:** Analysis of molecular variance (AMOVA) for two mangrove species after 10,000 permutations using ARLEQUIN 3.5

**Mangrove species**	**Microsatellite**				**cpDNA**			
	**Variance**	**% total**	***F***_***ST***_	***P***	**Variance**	**% total**	***F***_***ST***_	***P***
***Avicennia germinans***								
Among estuaries	1.113	30.39	0.304	**0.000**	2.345	66.80	0.668	**0.000**
Within Estuaries	2.550	69.61			1.165	33.20		
***Rhizophora mangle***								
Among estuaries	0.819	35.99	0.399	**0.000**	2.875	56.23	0.562	**0.000**
Within Estuaries	1.229	64.01			2.238	43.77		

Analysis of species *Avicennia germinans* (black mangrove) and *Rhizophora mangle* (red mangrove) was performed comparing four estuaries of Panama based on microsatellite and cpDNA polymorphism.

**Table 3:** **Pairwise genetic structure for two mangrove species across four estuaries in Panama using *F***_***ST***_

		**Caribbean**		**Pacific**	
***Avicennia germinans***		Bocas del Toro	Costa Arriba	Montijo Gulf	San Miguel Gulf
**Caribbean**	Bocas del Toro	-	0.149*	0.817*	0.721*
	Costa Arriba	0.068*	-	0.740*	0.658*
		0.063 (0.035 - 0.099)			
**Pacific**	Montijo Gulf	0.403*	0.311*	-	0.528*
		0.372 (0.298 - 0.453)	0.287 (0.214 - 0.367)		
	San Miguel Gulf	0.461*	0.369*	0.134*	*-*
		0.442 (0.340 - 0.549)	0.361 (0.268 - 0.460)	0.140 (0.083 - 0.201)	
***Rhizophora mangle***					
**Caribbean**	Bocas del Toro	-	*0.000*	0.706*	0.504*
	Costa Arriba	−0.008	-	0.706*	0.504*
		0.011 (0.003 - 0.019)			
**Pacific**	Montijo Gulf	0.471*	0.490*	-	0.353*
		0.453 (0.327 - 0.546)	0.460 (0.334 - 0.553)		
	San Miguel Gulf	0.494*	0.512*	0.078*	-
		0.470 (0.383 - 0.555)	0.479 (0.388 - 0.564)	0.071 (0.046 - 0.104)	

The AMOVA was performed using 10,000 permutations in ARLEQUIN 3.5. It is indicated the *F*_*ST*_ values derived from both microsatellite (below the diagonal) and cpDNA data (above the diagonal and in italics) for each pairwise comparison across estuaries. The asterkisk indicate P < 0.05, after Bonferroni corrections. Underlined values represent the unbiased *F*_*ST*_ estimates following the *ENA* method that correct by the presence of null alleles on *F*_*ST*_ estimation (95% CI after 10,000 replicates).

In spite of these similar strong structures across oceans and estuaries, both species showed differences in how genetic diversity is subdivided between estuaries from the same coast. Pairwise *F*_*ST*_ indicates that population differentiation is lower between estuaries in the Caribbean coast than between estuaries in the Pacific coast (i.e. *F*_*ST*_= 0.07 and *F*_*ST*_ = 0.008 between two Caribbean estuaries and *F*_*ST*_*=* 0.134 and *F*_*ST*_ = 0.08 between two Pacific estuaries for *A. germinans* and *R. mangle* respectively).

The Bayesian structure analysis supports differences in how structure is organized at two levels (2) Between estuaries from the same coastal line and (3) within estuaries. *Avicennia germinans*, with the exception of the Costa Arriba estuary, shows evidence of two demes within each one of the other three estuaries (Figure 1 and Additional file 5). In comparison, *R. mangle* showed evidence of three demes within each one of the two Pacific estuaries but only one extended population across Caribbean coastline without any differentiation between Bocas del Toro and Costa Arriba estuaries and without any substructure within each one of these two estuaries (Figure 1 and Additional file 6).

**Figure 3:**
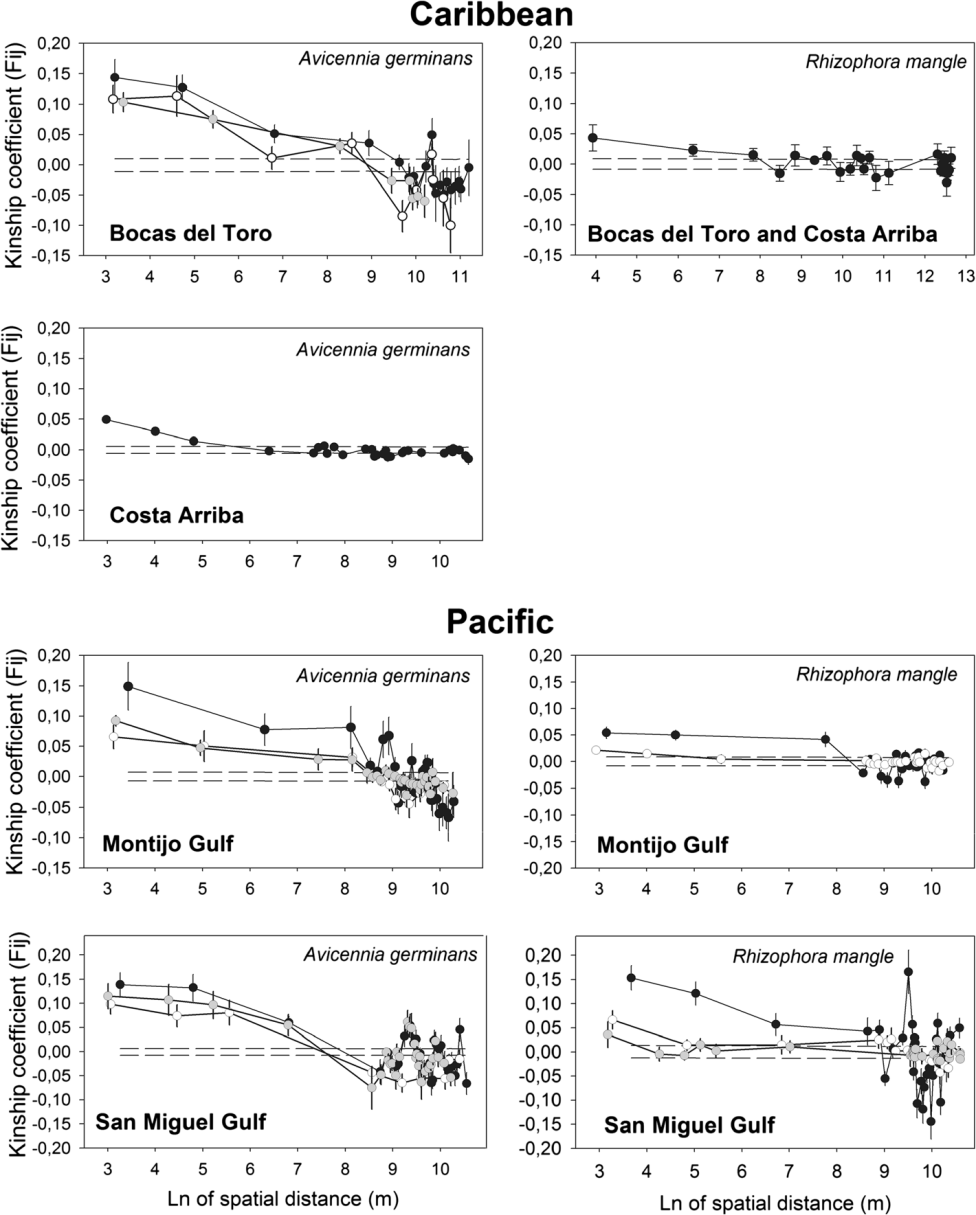
**Spatial autocorrelation of average Kinship coefficients (*F***_***ij***_**) against the natural logarithm of spatial distance inside four estuaries in Panama.** The Kinship-curve for the whole estuary is represented in black. Where GENELAND detected internal substructure, Kinship-curves for each genetic pool were calculated when N > 50 (white and grey curves). Dashed lines represent a 95% confidence interval around the hypothesis of no genetic structure for the whole estuary based on 10,000 permutations. We generated uneven lags with constant number of individuals inside distance classes (N > 100) with > 90% of pairwise relationships among nearest neighbors included within the first interval.

At level (3) within estuaries, the two mangrove species showed statistically significant negative slopes (*b*_*ld*_) in the kinship-distance curves (Figure 3 and Table 4). The strength of spatial genetic structure (SGS) measured by *Sp* statistics for the whole estuary is identical if we compare it with *Sp* of each deme inferred within the estuary. The exception was the Montijo Gulf estuary for both *A. germinans* and *R. mangle*. In both cases, *Sp* calculated for whole estuary was higher than when *Sp* was calculated for each deme within that estuary. In spite of that, we did not find differences in *Sp* values across seven *A. germinans*’s demes distributed in the four estuaries (Kruskall-Wallis ANOVA, P = 0.479). However, one of the *R. mangle*’s demes localized in San Miguel Gulf (*Sp*=0.04 ± SE 0.002) showed a stronger SGS compared to only one *R. mangle* deme shared between estuaries from Caribbean coastal line (*Sp* =0.01 ± SE 0.004) (Kruskall-Wallis ANOVA, P = 0.019) (Figure 1, Figure 3 and Table 4).

**Table 4:** Spatial Genetic Structure (SGS) parameters for two mangrove species across four estuaries in Panama

**Mangrove species/ Ocean**	**Estuary**	**N**	**b**_**ld**_	**F**_**A**_	***Sp***
			**(R**^**2**^**ld)**	**(SE)**	**(SE)**
*Avicennia germinans*					
Caribbean	Bocas del Toro	150	−0.0217*** (0.0639)	0.144*** (0.030)	0.025^a^ (0.007)
	Bocas del Toro (P)	76	−0.0330*** (0.1038)	0.108*** (0.023)	0.037^a^ (0.009)
	Bocas del Toro (MG )	72	−0.0145*** (0.4050)	0.104*** (0.016)	0.016^a^ (0.006)
	Costa Arriba (O)	241	0.0081*** (0.0222)	0.049*** (0.006)	0.008 (0.001)
Pacific	Montijo Gulf	307	−0.0256*** (0.0853)	0.149*** (0.039)	0.030^a^ (0.010)
	Montijo Gulf (Y)	85	−0.0147*** (0.0602)	0.065*** (0.020)	0.016^b^ (0.005)
	Montijo Gulf (P)	222	−0.0150*** (0.0370)	0.092*** (0.009)	0.017^ab^ (0.004)
	San Miguel Gulf	282	−0.0308*** (0.1919)	0.139*** (0.025)	0.036^a^ (0.007)
	San Miguel Gulf (LL)	80	−0.0280*** (0.2058)	0.098*** (0.020)	0.031^a^ (0.011)
	San Miguel Gulf ( TG )	202	−0.0271*** (0.1454)	0.115*** (0.026)	0.031^a^ (0.006)
*Rhizophora mangle*					
Caribbean	Bocas del Toro and Costa Arriba (O)	422	−0.0095*** (0.0039)	0.043*** (0.022)	0.010 (0.004)
Pacific	Montijo Gulf	281	−0.0131*** (0.0241)	0.054*** (0.011)	0.014^a^ (0.003)
	Montijo Gulf (TG)	37	-	-	-
	Montijo Gulf (P)	233	−0.0033** (0.0026)	0.021** (0.006)	0.003^b^ (0.001)
	Montijo Gulf (Y)	10	-	-	-
	San Miguel Gulf	237	−0.0276*** (0.0579)	0.154*** (0.025)	0.033^a^ (0.008)
	San Miguel Gulf (R)	41	-	-	-
	San Miguel Gulf (TG)	71	−0.0000108	0.066*** (0.019)	0.004^a^ (0.002)
	San Miguel Gulf (LL)	125	−0.00002175	0.036** (0.027)	0.008^a^ (0.011)

It is indicated the slopes (*b*_*ld*_) of the regression of kinship coefficient values on the natural logarithm of distance (*dl*_*ij*)_ for each estuary, the coefficient of determination R^2^, the average and standard error (SE) of the kinship coefficient among individuals separated by less than 100 m (i.e. 0.1 km) (F_A_) for each estuary, and the intensity of SGS (*Sp*) calculated for pairwise distance among individuals up to 10 Km within each estuary is shown. When GENELAND detected spatial genetic discontinuities within estuaries, the SGS parameters were recalculated for each genetic cluster when N > 50. Each one of the genetic clusters were named based on color assignation from Figure 1 including pink (P), malachite green (MG) and orange (O) within Caribbean estuaries and yellow (Y), purple (P), tourmaline green (TG), lapis lazuli (LL) and red (R) in Pacific estuaries.

***P<0.001; **P<0.01; *P<0.05.

The extent of SGS of two mangrove species was also different. *Avicennia germinans* showed a range of kinship values almost ten times higher than *R. mangle*. SGS was greater in *A. germinans* (*Sp* = 0.0186 ± SE 0.0026, N_demes_ = 7) than for *R. mangle* (*Sp* = 0.0019 ± SE 0.0031, N_demes_ = 4) across demes with N > 50 on scales up to 10 km (Kruskall-Wallis ANOVA, P = 0.0000) (Figure 3 and Table 4).

### Hierarchical structure of genetic variation in cpDNA

*Avicennia germinans* has lower genetic structure at the cpDNA level (*G*_*ST*_ = 0.35) relative to *R. mangle* (*G*_*ST*_ = 0.85) (Table 1). However, hierarchical organization of genetic structure of the two species at cpDNA shows more similar patterns than those inferred by nuclear microsatellites. In *A. germinans*, both Caribbean and Pacific coast samples showed strong structure between estuaries, (i.e. *F*_ST_ = 0.149 between Bocas del Toro and Costa Arriba estuaries in the Caribbean, and *F*_ST_ = 0.528 between Montijo Gulf and San Miguel Gulf in the Pacific). However, some haplotypes were shared between estuaries along same coastline but separated by ~300 km. In contrast, all *R. mangle* individuals shared a single haplotype on the Caribbean coast, while on the Pacific side although both estuaries shared the same two haplotypes; strong genetic population structure existed between Montijo Gulf and San Miguel Gulf (i.e. *F*_ST_ = 0.353) (Table 2 and Figure 2). Most of the genetic variation in cpDNA genome is partitioned among ocean basins, with 67% for *A. germinans* and 33% of the variation partitioned within estuaries (Table 2 and Additional file 2). *Rhizophora mangle* shows a similar pattern with 56% of the variation partitioned among oceans and 44% within estuaries (Table 2). Contrary to what was observed across microsatellites, the SGS analysis based on cpDNA did not show significant slopes within each one of the four Panamanian estuaries analyzed, perhaps due to limited sample size (Additional file 3).

### Comparison of pollen and seed migration rates

In *A. germinans* the pollen-to-seed gene flow ratio was negative, or basically zero (*r* = −0.64), which indicates one to two times higher gene flow via hydrochorious seed dispersal than entomophilous dispersed pollen gene flow. In comparison, the ratio obtained in *R. mangle* indicates approximately seven times higher ambophilous pollen gene flow than hydrochorious seed gene flow (*r* = 7.65). However, because the confidence limits of each species overlap with the expected value of equal seed and pollen flow, we could not reject the null hypothesis of *m*_*pollen*_ = *m*_*seed*_ gene flow in either species (Table 1).

## Discussion

Mangrove communities are critically important ecosystems that are high in aquatic and terrestrial biological diversity in tropical and subtropical ecosystems worldwide [2,5]. Today they are threatened by high rates of anthropogenic disturbance, including habitat destruction, pollution, fragmentation, and changes in oceanic and estuarine environments due to climate change [9,10,34]. The goal of this study was to document spatial genetic structure of two dominant Neotropical mangrove species at three spatial levels (1) among four estuaries in Panama (2) Between two estuaries from same coastal line and (3) within each one of the four estuaries. These data provide critical information to understanding how genetic diversity is structured and maintained within mangrove species and communities.

Both mangrove species showed a strong genetic break across the CAI. However, the patterns of diversity observed in this study were the opposite of what we had expected for both species. We found significant differences between estuaries from same coastline and also IBD within estuaries in both mangrove species. In addition, both mangrove species showed comparable outcrossing rates, contrary to other reports on their mating systems. Further, *A. germinans* showed much higher levels of genetic diversity, especially in plastid genomes, than *R. mangle*, in spite of the fact that *R. mangle* populations have a more continuous distribution. Finally, although there is documented evidence for extreme LDD in both mangrove species, our evidence mostly indicates restricted gene dispersal overall and largely equivalent rates of seed and pollen dispersal. Below, we interpret the observed population genetic differences of these species as well as the combined effects of species’ life histories, gene dispersal limitation, and biogeographic history.

### The ecological imprint on genetic structure: mating system and Pollen vs. seed movement

The two mangrove species showed strong genetic structure across four estuaries analyzed, including evidence of substructure and IBD within estuaries. Nevertheless, the patterns of genetic structure were very different between species. Microsatellites revealed lower gene diversity and lower genetic structure in *R. mangle* than *A. germinans*. This contradicts the predictions based upon an outcrossing mating system where assumed outcrossing species, such as *A. germinans,* are expected to have lower genetic structure than the mixed-mating *R. mangle*[33,35].

The protandry reported in *A. germinans* is the main evidence that supports an outcrossing mating system in this species because the flower-developing mechanism makes autogamy unlikely (i.e. within-flower pollination) [24]. However, absence of self-pollination (autogamy) is not equivalent to self-incompatibility because pollination could ocurr between different flowers on the same plant (geitonogamy). Other *Avicennia* species from Indo-West Pacific region including *A. marina* and *A. officinalis* show this pattern [36,37]. There is no direct evidence of self-incompatibility in *A. germinans* that we know of. It is possible that the patchy spatial distribution of *A. germinans* populations observed in Panama has an effect on the levels of outcrossing and therefore on the genetic structure in this species. In isolated individuals or low density populations geitonogamy could be an advantageous breeding system [36]. Moreover, the *r*=−0.64 estimates of pollen vs. seed flow suggests that although seed dispersal is likely equivalent to pollen dispersal in *A. germinans*, in a patchy matrix, this seed dispersal still is usually very local, leading to greater biparental inbreeding, and increasing population genetic differentiation and spatial genetic structure in nuclear genomes [38,39].

Our data also suggest that historically ambophilous pollen dispersal mechanism of *R. mangle* has been more efficient promoting outcrossing and long-distance gene flow than entomophilous pollination system of *A. germinans.* Based on previous genetic studies, it is predicted to have higher dispersal potential and therefore low genetic structure in species with wind pollination system over species with insect pollinator system [33]. However, reproductive biology studies in other ambophilous species suggest a completely opposite trend indicating that in self-compatible species as is the case of *R. mangle*, wind is actually the mechanism that promotes selfing and that out-crossing is associated with insect pollen distribution. The reason is that abiotic mechisms as wind do not target distant receptive flowers as efficiently as insects [40,41].

Although we do not know the rates of wind-to-insect pollen dispersal in *R. mangle*, the ratios of pollen-to-seed dispersal of r=7.7 estimated in this study are lower than those estimated in exclusively wind-dispersed plants (r=17 [42] and r=200 [43]). In addition, based on the Hamilton and Miller’s method calculations, pollen vs. seed was not significantly different from each other meaning that ambophilous pollen dispersal is less efficient than exclusively anemophilous pollen dispersal and/or that seed dispersal in this species is comparatively higher than in other exclusively wind pollinated species.

The contrast of seed viability between the two species may also have a strong influence in levels of genetic structure. *Rhizophora mangle* species have the highest longevity seeds of any mangrove genus [15,29]. Direct experiments regarding establishment of seeds after long periods of floating exposure in sea water have showed a 60% success rate after 247 days floating for *R. mangle*, higher than any other Rhizophoracea species [44]. There is no similar quantitative data on establishment success after dispersal for *A. germinans*; however, its seed longevity is shorter than *R. mangle*[29]. The local genetic structure observed in Panama largely corroborates this pattern, especially in the Caribbean, where two estuaries separated by 300 km showed identical chloroplast and nuclear genetic diversity in *R. mangle* but strong structure in *A. germinans*. Although *A. germinans* showed evidence of shared cpDNA haplotypes among estuaries on the same coast, there are also some cpDNA haplotypes and nuclear alleles that are restricted to each estuary, generating strong structure, even within estuaries. Thus, in a variable estuarine environment where seed movement is stochastic [18], our data suggests that higher propagule longevity leads to a greater chance of successful establishing at long distances, increasing gene flow and decreasing population structure [45,46].

### The historical imprint on genetic structure

The Isthmus of Panama represents a 20 My to three My old barrier to seed gene flow between the Atlantic and Pacific oceans, and is the narrowest terrestrial area in the New World that separates mangrove populations in each ocean [47,48]. Based on microsatellite and chloroplast genetic diversity observed across the Isthmus, this land mass has created high levels of genetic structure and a strong barrier for contemporary seed gene flow. Moreover, our results indicate that the Isthmus also represents a strong barrier to pollen flow in each species. This is likely due to the absence of a continuous terrestrial population that spans the entire distance between coasts. Thus, the strong isolating effect that the rise of the CAI has had on population differentiation for these two and other species highlights the role of restricted seed dispersal in creating spatial genetic structure in hydrochorious marine species [49-51].

Population differentiation observed between different oceans is exceptionally high compared to other tropical tree species. For example, Dick and Huertz [52] report an average *F*_*ST*_= 0.14 for microsatellites variation from the Neotropical tree *Symphonia globulifera.* Within Panama, *S. globulifera* averaged *F*_*ST*_ = 0.11 for samples taken across the Isthmus of Panama, which is within the range of observations that we see when comparisons are made within oceans for the mangrove species here. However, even trans-Andean *F*_*ST*_ between Mesoamerican populations of bird pollinated, mammal dispersal *S. globulifera* and those in the Amazon separated by > 3,000 km showed maximum *F*_*ST*_ = 0.27, still lower than lowest pairwise *F*_*ST*_ for *A. germinans* made between trans-Isthmian populations of Montijo Gulf and Costa Arriba (*F*_*ST*_ = 0.31) separated by < 100 km. Furthermore, populations of insect pollinated and wind dispersed mahogany (*Sweitenia macrophyla*) across Mesoamerica also show a lower overall *F*_*ST*_ = 0.10, with the largest pairwise differences (*F*_*ST*_*=* 0.238) observed between Panamanian and Guatemalan populations at a distance of > 1,600 km [53].

In spite of similar effects of CAI in the genetic structure of these two mangroves, we found unexpectedly high levels of cpDNA diversity and structure in *A. germinans* that suggests a level of diversity that could be more influenced by population history and demography than current gene flow [54]. The cpDNA diversity observed in *A. germinans* is remarkable given the small sample size and short geographic distances separating the populations both within and between coasts. This high level of diversity could be indicative of historical processes determining spatial genetic structure of populations. Although the fossil record of mangroves in Pleistocene is scarce and therefore the reconstruction of current mangrove distribution is very speculative, several lines of evidence suggest that mangrove ecosystem were under episodic crises during the Quaternary, specially associated to sea-level and temperature/humidity fluctuations [55-57]. In particular, it is possible that current populations of *A. germinans* represent remnants of refugial populations created during the Pleistocene [58]. In fact, Panamanian populations are genetically diverse compared to other regions, and it has been suggested that both ancient introgressive hybridization and secondary contact between *A. germinans* and its sister species *A. bicolor* has occurred in Panama (especially on the Pacific side), generating a hotspot of genetic diversity [31].

Our results in *A. germinans* contrast strongly with *R. mangle*, where very little cpDNA diversity was observed. These two species differ in their density and distribution, with *R. mangle* forming extremely dense, continuous forests near to shore, and *A. germinans* forming patchily distributed, lower-density stands at low and middle intertidal zones. One hypothesis that could explain the current distribution of cpDNA diversity is that mangrove populations represent relicts of much larger ancestral populations but that after Pleistocene-Holocene sea-level fluctuations, the mangrove composition shifted to a *R. mangle* -dominated community [55,57,59]. Under this scenario, *R. mangle* could have resulted in a more efficient colonizer than *A. germinans* follow a stepping-stone dispersion pattern. This process combined with self-fertilization observed in *R. mangle* could be the responsible of the current continous and dense populations and the low cpDNA and nuclear diversity observed in this species [57]. Alternatively, it is also possible that only certain older lineages of *R. mangle* that are well adapted to current conditions survived in the Pleistocene-Holocene sea-level fluctiations and that only those exclusive lineages recolonized available habitats during the Holocene or that longer time of *R. mangle* presence in neotropics generated more lost of diversity via genetic drift than younger *A. germinans*.

Regardless of the explanation, *A. germinans* joins the ranks of tropical tree species in Panama that show complex biogeographic history with disproportionately high levels of cpDNA diversity relative to other parts of their range [60]. The historical complexity of Panama was also evident when we analyzed the geographical variation in structure with greater population differentiation in Pacific versus Caribbean estuaries for both species in nuclear and plastid genetic markers. In the case of *R. mangle*, introgressive hybridization between *R. mangle* and its sister species, *R. racemosa*, is a novel source of genetic variation exclusive to the Pacific [13]. Thus, the levels of genetic structures and patterns of biodiversity in *R. mangle* are completely different between Caribbean and Pacific due to independent historical processes in the occurrence and sympatry of *R. mangle* and *R. racemosa*.

### The influence of coastal morphology

Hydrochory *per se* is assumed to be one of the most efficient mechanisms for LDD in plants. Therefore, it is expected that hydrochorious plant species should have high gene flow among populations and low genetic structure, especially in local geographic areas [61-63]. Our data contradict this hypothesis because the two mangrove species resulted genetically structured among and within estuaries, indicating local restrictions to both pollen and seed dispersal, especially in *A. germinans* species. Currently, one of the major threats in mangroves is fragmentation and sea-level changes associated with climate changes [10,11]. Historically, both mangrove species have experimented sea-level fluctuations at several times and climate changes [3,4,55,56], thus, our results suggest that historically *R. mangle* have maintained more gene dispersal of both pollen and seed dispersal than *A. germinans.* However*,* our results also suggested that geographic location is important in predicting levels of genetic structure in mangroves. Pacific populations proved to be more structured than Caribbean populations in both mangrove species. One possible explanation is biological. The Pacific coast has been characterized by ancient hybridization between *A. germinans* and *A. bicolor*, but there is also the current scenario of introgressive hybridization between *R. mangle* and *R. racemosa*. Both hybridization processes are apparently complicating current levels of structure compared with Caribbean populations [13,31]. The other possible explanation is abiotic, including basin geomorphology and connectivity due to ocean currents. Our results suggest that current or historical landscape characteristics of Pacific estuaries are in some way enhancing pollen and seed dispersal limitations, generating more structure compared to Caribbean estuaries. Similarly, the density of *A. germinans* is very variable and in some places, for example in French Guiana *A. germinans* could be more dense and extended than *R. mangle*[64]. In consequence, patterns of genetic structure among and within estuaries in that region could be completely different to observed in Panama. Thus, although life-history traits are important to predict expected genetic structure, landscape settings are generating a variety of situations on local scales that complicate any prediction in terms of expected levels of genetic structure [20]. Therefore, although long distance gene flow between South America and West Africa has been observed in both *A. germinans* and *R. mangle*[12,13], estuarine geomorphology and ocean currents in Panama, especially on the Pacific side, seem to be more complex, preventing pollen and seed dispersal.

## Methods

### Study sites and sampling strategy

We collected leaf samples from *A. germinans* and *R. mangle* trees within the four largest estuaries in Panama: Bocas del Toro and Costa Arriba in the Caribbean and Montijo Gulf and San Miguel Gulf in the Pacific (Figure 1). Within each estuary, we selected ten equidistant sites across the geographic contour for sampling. The sampling sites included rivers, streams, channels and shoreline areas, each with riverine or fringe mangrove forests. Within each sampling site we established transects parallel to the water's edge, and randomly selected a maximum of 30 adult trees for each mangrove species with a dbh ≥ 10 cm and spaced with a minimum distance of 5 m between sampled trees. All selected trees were geo-referenced using a GPS or a compass and measuring tape.

### Microsatellite analysis

We used 11 and 6 microsatellite loci for the analysis of 980 *A. germinans* individuals and 940 *R. mangle* individuals respectively, following established protocols [13,65-67] but with modifications [68] (See Additional file 4 for details). We calculated the average number of alleles per locus, observed heterozygosity (*H*_*o*_), expected heterozygosity (*H*_*e*_), and the fixation index (*F*_*IS*_) for each species across the four estuaries using GENALEX 6.0 [69]. In addition, we calculated the outcrossing rate by hand as 1 = *F*_*IS*_/1 + *F*_*IS*_[70]. For each locus, we tested deviations from Hardy-Weinberg equilibrium (HWE) and linkage equilibrium (LE) with GENEPOP 3.4 [71]. Also, we tested for the presence of null alleles and scoring problems associated with allelic stuttering or allelic dropout using MICROCHECKER 2.2.3 [72]. This analysis found no stutter bands or genotyping errors across microsatellites. However, some loci showed a positive presence of null alleles, ranged from −0.04 to 0.26 in *A. germinans* and from −0.18 to 0.29 in *R. mangle* (Additional File 4).

We compared the hierarchical genetic structure of *A. germinans* and *R. mangle* at three geographic levels: (1) Among four estuaries, (2) Between two estuaries along same coastline and (3) Within estuaries. For (1) among estuaries, we used a *F*_*ST*_ based AMOVA [73] in ARLEQUIN 3.5 [74] after 10,000 permutations. We compare this result with the *ENA* method implemented in *FREENA*[75] to correct the bias induced by the presence of null alleles on the *F*_*ST*_ estimation after 10,000 replicates (Additional File 4). For levels (2) Between two estuaries along same coastline and (3) Within estuaries we used STRUCTURE 2.2 [76-78], GENELAND 3.1.4 [79,80] and, for mixed-mated *R. mangle*, INSTRUCT [81] .

In STRUCTURE 2.2 we assumed an admixed model and a uniform prior probability of the number of populations, *K*. All the runs were performed with 500,000 MCMC replicates after a burn-in of 50,000 replicates. We used a model of correlated allele frequencies varying the level of structure from *K* = 1 to 6 populations. Ten independent runs were done for each value of *K* to generate our estimate of the true number of demes [82]. Previous empirical and simulation analysis using STRUCTURE showed that null allele presence have a low effect in the accuracy of assignment tests [83,84]. This effect was moderate even in populations with a frequency of null alleles > 0.917 for a single locus [83]. Therefore we performed the STRUCTURE 2.2. analysis with no correction to the raw data to account for null alleles. Nevertheless, the GENELAND 3.1.4. analysis was performed using a explicit null alleles presence model as we explain below.

The GENELAND 3.1.4 model assumes that population membership is structured across space. Thus, if this assumption is correct, the power of inferring clusters based upon the combination of genetic and geographical information increases compared with using STRUCTURE alone [85,86]. However, in the case of weak spatial organization, the inferred structure of GENELAND is expected to be similar to STRUCTURE inference [80]. For each run in GENELAND, we used the spatial D-model to calculate allele frequencies and set the maximum rate of the Poisson process and the maximum number of nuclei (i.e. three times the total number of individuals) according to the total number of individuals collected within each coast. In addition, we set an uncertainty attached to spatial coordinates fixed to 50 m. We performed ten independent runs of GENELAND allowing *K* to vary from 1–10 populations using the simultaneously uncorrelated allele frequency model, the spatial model, and the null allele model due to the presence of null alleles detected in both mangrove species (Additional File 4). We completed 100,000 iterations for each independent run, saving every 10^th^ iteration using a burn-in of 5,000 iterations. Finally, as mating systems may be different between the two species under study and selfing rates could influence deme structure in mixed-mating species, we used INSTRUCT to infer simultaneously the selfing rates of *R. mangle* and its genetic structure at two levels: (2) Between two estuaries along same coastline and (3) Within estuaries. In INSTRUCT, HWE is not assumed as it is in STRUCTURE; rather the expected genotype frequencies are calculated based on selfing rates. We ran five independent chains, each chain having 500,000 iterations steps, 250,000 burn-in iterations, a thinning interval of 10 and assuming a Dirichlet process of mixture.

### Spatial genetic structure within estuaries

We investigated spatial genetic structure (SGS) at level (3) within estuaries using a spatial autocorrelation analysis [87]. For this analysis, we calculated the pairwise kinship coefficient between all individuals (*F*_*ij*_) separated at different distance classes following [88] up to 10 km. Kinship coefficients (*F*_*ij*_) were regressed using the logarithm of the spatial distance between individuals (*ld*_*ij*_) within each one of the estuaries analyzed. Standard errors were assessed by jackknifing data over each locus. We generated uneven lags with constant number of individuals inside distance classes (N > 100) with > 90% of pairwise relationships among nearest neighbors included within the first interval. Using SPAGeDi 1.3 [89], we calculated the regressions of kinship coefficients (*F*_*ij*_) vs. natural logarithm of distance classes (*ld*_*ij*_) to provide the regression slope *(b*_*ld*_). We tested the significance of SGS and the IBD in two dimensions using the observed slope (*b*_*ld*_) of the linear regression of the kinship coefficient on the logarithm of the distance class against the null hypothesis H_o_: *b*_*ld*_ = 0 (i.e. the overall absence of SGS) by comparing the observed values with those obtained after 10,000 permutations of individuals between locations. Where GENELAND detected a spatial deme within an estuary, this procedure was applied to each inferred deme that was represented by at least 50 individuals, excluding admixed individuals.

To compare the extent of SGS between the two mangrove species over the same geographic scales and across different estuaries, we calculated the *Sp* statistics on spatial scales of up to 10 km. The *Sp* statistics were calculated from the slope of the regression *(b*_*ld*_) of the Kinship coefficient [Fij of 88] against the logarithm of the distance, *S*_*p*_ = − *b*_*l*10*km*_/(1 − *F*_*A*_) where the regression slope, *b*_*l10km*_^*,*^ is less than 10 km and *F*_*A*_ is the average kinship coefficient between individuals belonging to the first distance class (< 100 m in all cases). This first distance class included all pairs of neighbors [90]. In the cases where we found demes within estuaries we repeated this procedure for each one of the subpopulations if N > 50.

### Chloroplast analysis

A subsample of 58 individuals of *A. germinans* and 60 individuals of *R. mangle* and distributed across the four estuaries (i.e. 14–18 individuals per estuary) was sequenced using two chloroplast (cpDNA) non-coding regions *atpI-atpH* and *psbJ-petA*[91]. We redesigned primers to avoid short-repeat regions and improve sequence quality in all regions except for the *atpI-atpH* region in *A. germinans*[91]. Modified primers for the *psbJ-petA* region in *A. germinans* were F: AGATTGATCGATATCGGGTTC and R: GGAAAACCGAAACCCAGAC*.* Modified primers for *R. mangle* analysis, PCR and sequencing specifications for both species were described previously [13]. For each mangrove species we calculated the haplotype diversity using DNASP 4.5 [92]. In addition we constructed a median joining network [93] for the combined cpDNA regions using NETWORK 4.5.1.0 (fluxus-engineering.com). We calculated population structure using ARLEQUIN 3.5 [74] at level (1) among four estuaries and (2) between two estuaries from same coastal line. Finally, we used SPAGEDI 1.3 [89] to investigate SGS at level (3) within estuaries, using a spatial autocorrelation analysis of cpDNA variation. For this analysis, we calculated the pairwise kinship analogue coefficient between all individuals based on the genetic distances between haplotypes (*N*_*ij*_) separated at different distance classes vs. natural logarithm of distance classes (*ld*_*ij*_) to provide the regression slope *(b*_*ld*_) on spatial scales up to 10 km in a procedure similar to that used for microsatellites [89].

### Comparison of pollen and seed migration rates

In order to compare pollen and seeds migration rates across four estuaries we estimated *F*_*ST*_ and *F*_*IS*_ from microsatellite data and *G*_*ST*_ from cpDNA data using SPAGEDI 1.3 [89]. These *F*-estimates were used to calculate the ratio (r) of pollen migration (m_p_) to seed migration (m_s_) (i.e. m_p_/m_s_) for each mangrove species following the equation *r* = [(1/*F*_*ST bipar*_ − 1)(1 + *F*_*IS*_)] − 2(1/*G*_*ST mat*_ − 1)/(1/*G*_*ST mat*_ − 1) [Eq. 5a, 43]. In addition, the same *F*-statistics based on microsatellite and cpDNA variations were used to test the null hypothesis m_seed_ = m_pollen_ comparing the expected maternal *F*_*ST*_ predicted from microsatellite variation (i.e. biparental *F*_*ST*_) vs. the observed maternal *F*_*ST*_ estimated as *G*_*ST*_ from actual cpDNA variation [21]. The expected maternal *F*_*ST*_ was calculated following *F*_*ST mat*_ = (*a*_*bipar*_*F*_*ST bipar*_)/*a*_*mat*_ + (*a*_*bipar*_ − *a*_*mat*_)*F*_*ST bipar*_ where *a*_*mat*_ = 2.0 and *a*_*bipar*_ change depending of outcrossing rate (*t*) calculated for each species from microsatellite data [Eq. 10, 21]. Under this procedure, the null hypothesis is rejected if confidence intervals (± 2SE) of observed and expected values fail to overlap [21].

## Conclusions

Although there is documented direct and genetic evidence for extreme LDD in mangrove species *A. germinans* and *R. mangle*, our data across estuaries in Panama showed restricted gene dispersal overall and equivalent rates of seed and pollen dispersal in both mangrove species. *Rhizophora mangle* showed lower gene diversity and lower genetic structure than *A. germinans*. This suggest that an amphophilous pollen syndrome combined with a higher propagule longevity leads to a greater chance of successful establishing at long distances, increasing gene flow and decreasing gene diversity and population structure. However*,* species density, coastal geomorphology as well as ocean currents could vary across ocean basins and estuaries, generating a variety of situations on local scales that complicate any prediction in terms of expected levels of genetic structure.

## Competing interests

The authors declare that they have no competing interests.

## Authors' contributions

ICS, EB and WOM defined the research topic and the experimental design. ICS carried out the laboratory experiments and generate the data. ICS and AJ analyze data and wrote the manuscript. All authors have contributed to, seen and approved the manuscript.

## Supplementary Material

Additional file 1 **Genetic diversity of nuclear (i.e. microsatellites) and chloroplast genomes for *Avicennia germinans *(black mangrove) and *Rhizophora mangle *(red mangrove) in Panama.** The number of individuals analyzed per estuary, the number of alleles, observed heterocigosity (Ho), and the expected heterocigosity (He) of microsatellites calculated with GENALEX 6.0 is shown. In addition, the number of individuals analyzed per estuary, the number of haplotypes and the haplotype diversity found in chloroplast using DNASP 4.5 and NETWORK 4.5.1.0 (fluxus-engineering.com), and the GenBank accession number of each individual analyzed is indicated. Click here for file

Additional file 2 **Median joining network indicating and geographic distribution of cpDNA haplotypes found in *Avicennia germinans *(Black mangrove).** Within the network, the haplotype name and the number of individuals per each haplotype is indicated. In addition, for each estuary, the geographic distribution of haplotypes and their frequency (i.e. pie) is indicated. Click here for file

Additional file 3 **Median joining network indicating and geographic distribution of cpDNA haplotypes found in *Rhizophora mangle *(Red mangrove).** Within the network the haplotype name and the number of individuals per each haplotype is indicated. In addition, for each estuary the geographic distribution of haplotypes and their frequency (i.e. pie) is indicated. Click here for file

Additional file 4 **PCR conditions for 11 microsatellite loci for *A. germinans *(black mangrove) [**[65,66]**] and six microsatellite loci for *R. mangle *(red mangrove) [**[67]**] following established protocols [**[13,65,66]**] with modifications including three primers in the PCR thus: a dye tagged M13 universal forward primer (5’-CACGACGTTGTAAAACGAC-3’)[**[68]**]**, primer Forward (F) and primer Reverse (R) where either F or R primer (indicated with asterisk *) had a tail at the 5’ end that was identical to the M13 universal forward primer sequence. Amplified fragments from both mangrove species were electrophoretically separated on ABI 3130*xl* and analyzed using ABI PRISM® GeneMapper™ software version 3.7. For each estuary is indicated the frequency of null alleles calculated by the Brookfield method 1 in MICROCHECKER 2.2.3 [72]. Click here for file

Additional file 5 **Bayesian genetic assignment of *Avicennia germinans *(black mangrove) from two Caribbean (Bocas del Toro and Costa Arriba) and two Pacific (Montijo Gulf and San Miguel Gulf) estuaries in Panama based on STRUCTURE ver. 2.2 and GENELAND ver. 2.0.12.** The true K for each procedure after simulations is indicated. Click here for file

Additional file 6 **Bayesian genetic assignment of *Rhizophora mangle *(Red mangrove) from two Caribbean (Bocas del Toro and Costa Arriba) and two Pacific (Montijo Gulf and San Miguel Gulf) estuaries in Panama based on STRUCTURE ver. 2.2, INSTRUCT and GENELAND. 2.0.12.** The true K for each procedure after simulations is indicated. In addition, INSTRUCT was used to help to simultaneously infer the selfing rates of this mixed mating species and the demic structure on both sides of the Isthmus. Click here for file

## References

[B1] Twilley RR Hall CAS Properties of mangrove ecosystems related to the energy signature of coastal environments Maximum power: The ideas and Applications of H T Odum 1995 Niwot: University Press of Colorado 43 62

[B2] Tomlinson PB The botany of mangroves 1986 New York, New York, USA: Cambridge University Press

[B3] Graham A Diversification of gulf/caribbean mangrove communities through Cenozoic time Biotropica 1995 27 20 27 10.2307/2388899

[B4] Graham A Paleobotanical evidence and molecular data in reconstructing the historical phytogeography of Rhizophoraceae Ann Missouri Bot Gard 2006 93 325 334 10.3417/0026-6493(2006)93[325:PEAMDI]2.0.CO;2

[B5] Twilley RR Medina E Snedaker SC Yañez-Arancibia A Medina E Mooney HA, Cushman JH, Medina E, Sala OE, Schulze ED Biodiversity and ecosystem processes in tropical estuaries: Perspectives of mangrove ecosystems Functional Roles of Biodiversity: A global perspective 1996 New York: John Wiley & Sons Ltd 327 370

[B6] Twilley RR Messina MG, Conner WH Mangrove wetlands Southern forested wetlands ecology and management 1998 Boca Raton: Lewis Publishers 445 473

[B7] FAO The world's mangrove 1980–2005. FAO Forestry Paper 153 2007 Rome (Italy): Rome Food and Agriculture Organization of the United Nations

[B8] Ellison AM Mangrove restoration: Do we know enough? Restor Ecol 2000 8 219 229 10.1046/j.1526-100x.2000.80033.x

[B9] Valiela I Bowen JL York JK Mangrove forests: One of the world's threatened major tropical environments Bioscience 2001 51 807 815 10.1641/0006-3568(2001)051[0807:MFOOTW]2.0.CO;2

[B10] Alongi DM Present state and future of the world's mangrove forests Environ Conserv 2002 29 331 349

[B11] Alongi DM Mangrove forests: Resilience, protection from tsunamis, and responses to global climate change Estuarine Coastal Shelf Sci 2008 76 1 13 10.1016/j.ecss.2007.08.024

[B12] Nettel A Dodd RS Drifting propagules and receding swamps: Genetic footprints of mangrove recolonization and dispersal along tropical coasts Evolution 2007 61 958 971 10.1111/j.1558-5646.2007.00070.x 17439624

[B13] Cerón-Souza I Rivera-Ocasio E Medina E Jiménez JA McMillan WO Bermingham E Hybridization and introgression in New World red mangroves, *Rhizophora* (Rhizophoraceae) Am J Bot 2010 97 945 957 10.3732/ajb.0900172 21622465

[B14] Lema-Velez LF Polania J Urrego-Giraldo LE Dispersión y establecimiento de las especies de mangle del río Ranchería en el periodo de máxima fructificación Rev Acad Colomb Cienc 2003 27 93 103

[B15] Davis JH The ecology and geology role of mangroves in Florida. Papers from Tortugas lab 32 Carnegie Institute of Washington 1940 517 305 412

[B16] Sengupta R Middleton B Yan C Zuro M Hartman H Landscape characteristics of *Rhizophora mangle* forests and propagule deposition in coastal environments of Florida (USA) Landscape Ecol 2005 20 63 72 10.1007/s10980-004-0468-8

[B17] Gunn CR Dennis JV Tropical and temperate stranded seeds and fruits from the Gulf of Mexico Contrib Mar Sci 1973 17 111 121

[B18] Sousa WP Kennedy PG Mitchell BJ Ordonez LBM Supply-side ecology in mangroves: do propagule dispersal and seedling establishment explain forest structure? Ecol Monogr 2007 77 53 76 10.1890/05-1935

[B19] Dick C Hardy O Jones F Petit R Spatial scales of pollen and seed-mediated gene flow in tropical rain forest trees Trop Plant Biol 2008 1 20 33 10.1007/s12042-007-9006-6

[B20] Jordano P Pollen, seeds and genes: the movement ecology of plants Heredity 2010 105 329 330 10.1038/hdy.2010.28 20332803

[B21] Hamilton MB Miller JR Comparing relative rates of pollen and seed gene flow in the island model using nuclear and organelle measures of population Structure Genetics 2002 162 1897 1909 12524358 10.1093/genetics/162.4.1897 PMC1462371

[B22] Plaziat J-C Cavagnetto C Koeniguer J-C Baltzer F History and biogeography of the mangrove ecosystem, based on a critical reassessment of the paleontological record Wetlands Ecol Manage 2001 9 161 180 10.1023/A:1011118204434

[B23] Menezes MPM De Oliveira D De Mello CF Pollination of red mangrove, *Rhizophora mangle*, in Northern Brazil Acta Hortic 1996 437 431 434

[B24] Rathcke B Kass L Hunt RE Elliott NB, Edwards DC, Godfrey PJ Preliminary observations on plant reproductive biology in mangrove communities on San Salvador Island, Bahamas Proceedings of the Sixth Symposium on the Natural History of the Bahamas 1996 San Salvador, Bahamas: Bahamian Field Station Ltd 87 96

[B25] Lemus-Jiménez LJ Ramírez N Polinización y polinizadores en la vegetación de la planicie costera de Paraguaná, estado Falcón, Venezuela Acta Cient Venez 2003 54 97 114 14976781

[B26] Sánchez-Núñez DA Mancera-Pineda JE Pollination and fruit set in the main neotropical mangrove species from the Southwestern Caribbean Aquat Bot 2012 In press

[B27] Lowenfeld R Klekowski EJ Jr Mangrove genetics. I. Mating system and mutation rates of *Rhizophora mangle* in Florida and San Salvador Island, Bahamas Int J Plant Sci 1992 153 394 399 10.1086/297043

[B28] McKee KL Seedling recruitment patterns in a Belizean mangrove forest: effects of establishment ability and physico-chemical factors Oecologia 1995 101 448 460 10.1007/BF00329423 28306959

[B29] Rabinowitz D Dispersal properties of mangrove propagules Biotropica 1978 10 47 57 10.2307/2388105

[B30] Lovelock CE Feller IC McKee KL Thompson R Variation in mangrove forest structure and sediment characteristics in Bocas del Toro, Panama Caribb J Sci 2005 41 456 464

[B31] Nettel A Dodd RS Afzal-Rafii Z Tovilla-Hernandez C Genetic diversity enhanced by ancient introgression and secondary contact in East Pacific black mangroves Mol Ecol 2008 17 2680 2690 10.1111/j.1365-294X.2008.03766.x 18466233

[B32] Cerón-Souza I Toro-Perea N Cárdenas-Henao H Population genetic structure of Neotropical mangrove species on the Colombian Pacific coast: *Avicennia germinans* (Avicenniaceae) Biotropica 2005 37 258 265 10.1111/j.1744-7429.2005.00035.x

[B33] Loveless MD Hamrick JL Ecological determinants of genetic structure in plant populations Annu Rev Ecol Syst 1984 15 65 95 10.1146/annurev.es.15.110184.000433

[B34] Duke N Meynecke J Dittmann S Ellison A Anger K Berger U Cannicci S Diele K Ewel K Field C A world without mangroves Science 2007 317 41 42 17615322 10.1126/science.317.5834.41b

[B35] Hamrick J Response of forest trees to global environmental changes For Ecol Manag 2004 197 323 335 10.1016/j.foreco.2004.05.023

[B36] Clarke P Myerscough P Floral biology and reproductive phenology of *Avicennia marina* in south-eastern Australia Aust J Bot 1991 39 283 293 10.1071/BT9910283

[B37] Aluri J Observations on the floral biology of certain mangroves Proc Indian Natl Sci Acad Part B Biol Sci 1990 56 367 374

[B38] Kalisz S Nason J Hanzawa F Tonsor S Spatial population genetic structure in *Trillium grandiflorum*: the roles of dispersal, mating, history, and selection Evolution 2001 55 1560 1568 11580015 10.1111/j.0014-3820.2001.tb00675.x

[B39] Yu H Nason JD Ge X Zeng J Slatkin’s Paradox: when direct observation and realized gene flow disagree. A case study in Ficus Mol Ecol 2010 19 4441 4453 10.1111/j.1365-294X.2010.04777.x 20840599

[B40] Culley TM Weller SG Sakai AK The evolution of wind pollination in angiosperms Trends Ecol Evol 2002 17 361 369 10.1016/S0169-5347(02)02540-5

[B41] Dafni A Dukas R Insect and wind pollination in *Urginea maritima* (Liliaceae) Plant Syst Evol 1986 154 1 10 10.1007/BF00984864

[B42] Petit RJ Duminil J Fineschi S Hampe A Salvini D Vendramin GG Comparative organization of chloroplast, mitochondrial and nuclear diversity in plant populations Mol Ecol 2005 14 689 701 15723661 10.1111/j.1365-294X.2004.02410.x

[B43] Ennos RA Estimating the relative rates of pollen and seed migration among plant populations Heredity 1994 72 250 259 10.1038/hdy.1994.35

[B44] Steele OC Natural and anthropogenic biogeography of mangroves in the southwest Pacific Master Thesis 2006 USA: University of Hawai'i

[B45] Duke NC Genetic diversity, distributional barriers and rafting continents - more thoughts on the evolution of mangroves Hydrobiologia 1995 295 167 181 10.1007/BF00029124

[B46] Duke NC Ball MC Ellison JC Factors influencing biodiversity and distributional gradients in mangroves Global Ecol Biogeogr Lett 1998 7 27 47 10.2307/2997695

[B47] Coates AG Obando JA Jackson JBC, Budd AF, Coates AG The geologic evolution of the Central American isthmus Evolution and environment in Tropical America 1996 Chicago, Illinois, USA: University of Chicago Press 21 56

[B48] Montes C Cardona A McFadden R Morón SE Silva CA Restrepo-Moreno S Ramírez DA Hoyos N Wilson J Farris D Evidence for middle Eocene and younger land emergence in central Panama: Implications for Isthmus closure Geol Soc Am Bull 2012 In press7060577

[B49] Lessios HA The great american schism: Divergence of marine organisms after the rise of the Central American Isthmus Annu Rev Ecol Evol Syst 2008 39 63 91 10.1146/annurev.ecolsys.38.091206.095815

[B50] Miura O Torchin ME Bermingham E Molecular phylogenetics reveals differential divergence of coastal snails separated by the Isthmus of Panama Mol Phylogen Evol 2010 56 40 48 10.1016/j.ympev.2010.04.012 20399869

[B51] Knowlton N Weigt LA Solorzano LA Mills DK Bermingham E Divergence in proteins, mitochondrial-DNA, and reproductive compatibility across the Isthmus of Panama Science 1993 260 1629 1632 10.1126/science.8503007 8503007

[B52] Dick CW Heuertz M The complex biogeographic history of a widespread tropical tree species Evolution 2008 62 2760 2774 10.1111/j.1558-5646.2008.00506.x 18764917

[B53] Novick R Dick CW Lemes M Navarro C Caccone A Bermingham E Genetic structure of Mesoamerican populations of big-leaf mahogany (*Swietenia macrophylla*) inferred by microsatellite analysis Mol Ecol 2003 12 1885 2 10.1046/j.1365-294x.2003.01951.x 14629370

[B54] Austerlitz F Mariette S Machon N Gouyon P-H Godelle B Effects of colonization processes on genetic diversity: differences between annual Plants and tree Species Genetics 2000 154 1309 1321 10757772 10.1093/genetics/154.3.1309 PMC1461003

[B55] Woodroffe CD Grindrod J Mangrove biogeography: The role of Quaternary environmental and sea-level change J Biogeogr 1991 18 479 492 10.2307/2845685

[B56] van der Hammen T The Pleistocene changes of vegetation and climate in tropical South America J Biogeogr 1974 1 3 26 10.2307/3038066

[B57] Pil MW Boeger MRT Muschner VC Pie MR Ostrensky A Boeger WA Postglacial north–south expansion of populations of Rhizophora mangle (*Rhizophoraceae*) along the Brazilian coast revealed by microsatellite analysis Am J Bot 2011 98 1031 1039 10.3732/ajb.1000392 21653512

[B58] Petit RJ Aguinagalde I de Beaulieu J-L Bittkau C Brewer S Cheddadi R Ennos R Fineschi S Grivet D Lascoux M Glacial Refugia: Hotspots But Not Melting Pots of Genetic Diversity Science 2003 300 1563 1565 10.1126/science.1083264 12791991

[B59] Sandoval-Castro E Muñiz-Salazar R Enríquez-Paredes LM Riosmena-Rodríguez R Dodd RS Tovilla-Hernández C Arredondo-García MC Genetic population structure of red mangrove (*Rhizophora mangle* L.) along the northwestern coast of Mexico Aquat Bot 2012 99 20 26

[B60] Dick CW Abdul-Salim K Bermingham E Molecular systematic analysis reveals cryptic Tertiary diversification of a widespread tropical rain forest tree Am Nat 2003 162 691 703 10.1086/379795 14737707

[B61] Kudoh H Whigham DF Microgeographic genetic structure and gene flow in *Hibiscus moscheutos* (Malvaceae) populations Am J Bot 1997 84 1285 1293 10.2307/2446054 21708685

[B62] Ritland K Genetic structure, diversity, and inbreeding in the mountain monkey flower (*Mimulus caespitosus*) of the Washington Cascades Can J Bot 1989 67 2017 2024 10.1139/b89-255

[B63] Nilsson C Brown RL Jansson R Merritt DM The role of hydrochory in structuring riparian and wetland vegetation Biol Rev 2010 85 837 858 20233190 10.1111/j.1469-185X.2010.00129.x

[B64] Fromard F Vega C Proisy C Half a century of dynamic coastal change affecting mangrove shorelines of French Guiana. A case study based on remote sensing data analyses and field surveys Mar Geol 2004 208 265 280 10.1016/j.margeo.2004.04.018

[B65] Cerón-Souza I Rivera-Ocasio E Funk SM McMillan WO Development of six microsatellite loci for black mangrove (*Avicennia germinans*) Mol Ecol Notes 2006 6 692 694 10.1111/j.1471-8286.2006.01312.x

[B66] Nettel A Rafii F Dodd RS Characterization of microsatellite markers for the mangrove tree *Avicennia germinans* L. (Avicenniaceae) Mol Ecol Notes 2005 5 103 105 10.1111/j.1471-8286.2004.00851.x

[B67] Rosero-Galindo C Gaitan-Solis E Cardenas-Henao H Tohme J Toro-Perea N Polymorphic microsatellites in a mangrove species, *Rhizophora mangle* L (Rhizophoraceae) Mol Ecol Notes 2002 2 281 283 10.1046/j.1471-8286.2002.00232.x

[B68] Steffens DL Sutter SL Roemer SC An alternate universal forward primer for improved automated sequencing of M13 Biotechniques 1993 15 580 582 8251153

[B69] Peakall R Smouse PE GenAlEx 6: genetic analysis in Excel. Population genetic software for teaching and research Mol Ecol Notes 2006 6 288 295 10.1111/j.1471-8286.2005.01155.x PMC3463245 22820204

[B70] Weir BS Genetic data analysis II 1996 Sunderland, MA: Sinauer Associates, Inc.

[B71] Raymond M Rousset F GENEPOP Version 1.2.: population genetic software for exact test and ecumenicism J Hered 1995 86 248 249

[B72] Van Oosterhout C Hutchinson WF Willis DPM Shipley P MICRO-CHECKER: software for identifying and correcting genotyping errors in microsatellite data Mol Ecol Notes 2004 4 535 538 10.1111/j.1471-8286.2004.00684.x

[B73] Weir BS Cockerham CC Estimating F-statistics for the analysis of population structure Evolution 1984 38 1358 1370 10.2307/2408641 28563791

[B74] Excoffier L Lischer HEL Arlequin suite ver 3.5: a new series of programs to perform population genetics analyses under Linux and Windows Mol Ecol Resour 2010 10 564 567 10.1111/j.1755-0998.2010.02847.x 21565059

[B75] Chapuis M-P Estoup A Microsatellite null alleles and estimation of population differentiation Mol Biol Evol 2007 24 621 631 17150975 10.1093/molbev/msl191

[B76] Pritchard JK Stephens M Donnelly P Inference of population structure using multilocus genotype data Genetics 2000 155 945 959 10835412 10.1093/genetics/155.2.945 PMC1461096

[B77] Falush D Stephens M Pritchard JK Inference of population structure using multilocus genotype data: Linked loci and correlated allele frequencies Genetics 2003 164 1567 1587 12930761 10.1093/genetics/164.4.1567 PMC1462648

[B78] Falush D Stephens M Pritchard JK Inference of population structure using multilocus genotype data: dominant markers and null alleles Mol Ecol Notes 2007 7 574 578 10.1111/j.1471-8286.2007.01758.x 18784791 PMC1974779

[B79] Guillot G Mortier F Estoup A Geneland: a computer package for landscape genetics Mol Ecol Notes 2005 5 712 715 10.1111/j.1471-8286.2005.01031.x

[B80] Guillot G Estoup A Mortier F Cosson JF A Spatial Statistical Model for Landscape Genetics Genetics 2005 170 1261 1280 10.1534/genetics.104.033803 15520263 PMC1451194

[B81] Gao H Williamson S Bustamante CD A Markov Chain Monte Carlo approach for joint Inference of population structure and inbreeding rates from multilocus genotype data Genetics 2007 176 1635 1651 10.1534/genetics.107.072371 17483417 PMC1931536

[B82] Evanno G Regnaut S Goudet J Detecting the number of clusters of individuals using the software structure: a simulation study Mol Ecol 2005 14 2611 2620 10.1111/j.1365-294X.2005.02553.x 15969739

[B83] Carlsson J Effects of microsatellite null alleles on assignment testing J Hered 2008 99 616 623 10.1093/jhered/esn048 18535000

[B84] Hauser L Seamons TR Dauer M Naish KA Quinn TP An empirical verification of population assignment methods by marking and parentage data: hatchery and wild steelhead (*Oncorhynchus mykiss*) in Forks Creek, Washington, USA Mol Ecol 2006 15 3157 3173 10.1111/j.1365-294X.2006.03017.x 16968262

[B85] Coulon A Guillot G Cosson J-F Angibault JMA Aulagnier S Cargelutti B Galan M Hewison AJM Genetic structure is influenced by landscape features: empirical evidence from a roe deer population Mol Ecol 2006 15 1669 1679 10.1111/j.1365-294X.2006.02861.x 16629819

[B86] Hannelius U Salmela E Lappalainen T Guillot G Lindgren C von Dobeln U Lahermo P Kere J Population substructure in Finland and Sweden revealed by the use of spatial coordinates and a small number of unlinked autosomal SNPs BMC Genet 2008 9 54 18713460 10.1186/1471-2156-9-54 PMC2527025

[B87] Hardy OJ Vekemans X Isolation by distance in a continuous population: reconciliation between spatial autocorrelation analysis and population genetics models Heredity 1999 83 145 154 10.1046/j.1365-2540.1999.00558.x 10469202

[B88] Loiselle BA Sork VL Nason J Graham C Spatial genetic-structure of a tropical understory shrub, *Psychotria Officinalis* (Rubiaceae) Am J Bot 1995 82 1420 1425 10.2307/2445869

[B89] Hardy OJ Vekemans X SPAGeDi: A versatile computer program to analyse spatial genetic structure at the individual or population levels Mol Ecol Notes 2002 2 618 620 10.1046/j.1471-8286.2002.00305.x

[B90] Vekemans X Hardy OJ New insights from fine-scale spatial genetic structure analyses in plant populations Mol Ecol 2004 13 921 935 10.1046/j.1365-294X.2004.02076.x 15012766

[B91] Shaw J Lickey EB Schilling EE Small RL Comparison of whole chloroplast genome sequences to choose noncoding regions for phylogenetic studies in angiosperms: the tortoise and the hare III Am J Bot 2007 94 275 288 10.3732/ajb.94.3.275 21636401

[B92] Rozas J Sanchez-Delbarro JC Messeguer X Rozas R DnaSP, DNA polymorphism analyses by the coalescent and other methods Bioinformatics 2003 19 2496 2497 10.1093/bioinformatics/btg359 14668244

[B93] Bandelt HJ Forster P Rohl A Median-joining networks for inferring intraspecific phylogenies Mol Biol Evol 1999 16 37 48 10.1093/oxfordjournals.molbev.a026036 10331250

