# Supplementary figures and images for: Comparative genetic structure of two mangrove species in Caribbean and Pacific estuaries of Panama
Source: BMC Evol Biol. 2012 Oct 18;12:205. doi: 10.1186/1471-2148-12-205 (PMC3543234; doi:10.1186/1471-2148-12-205)

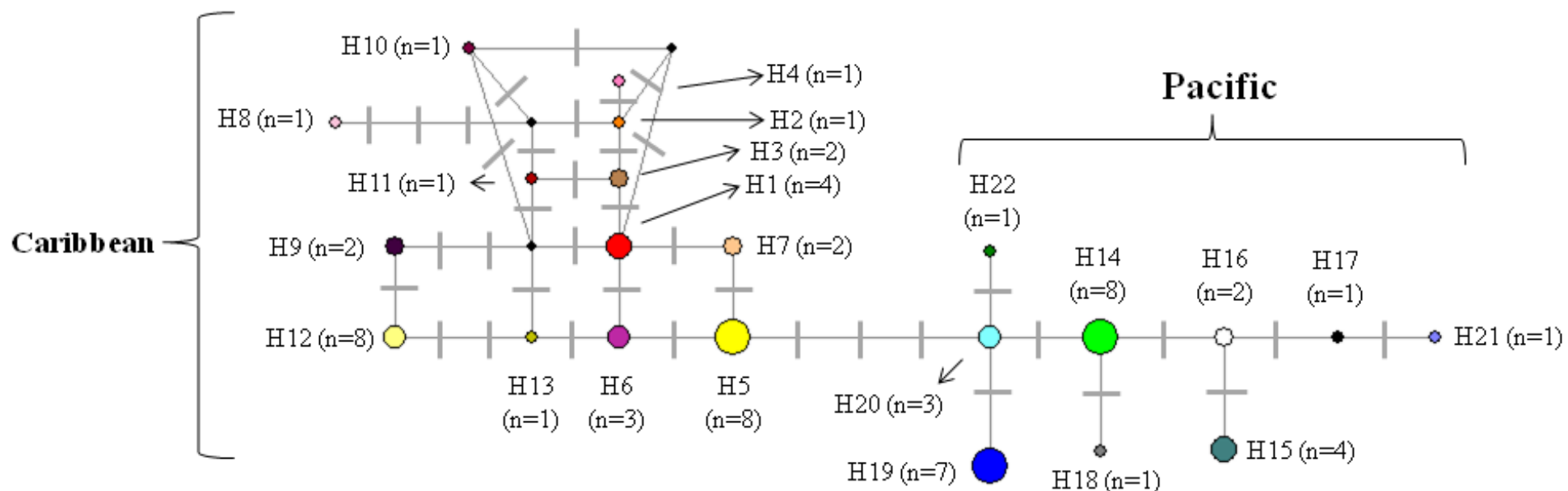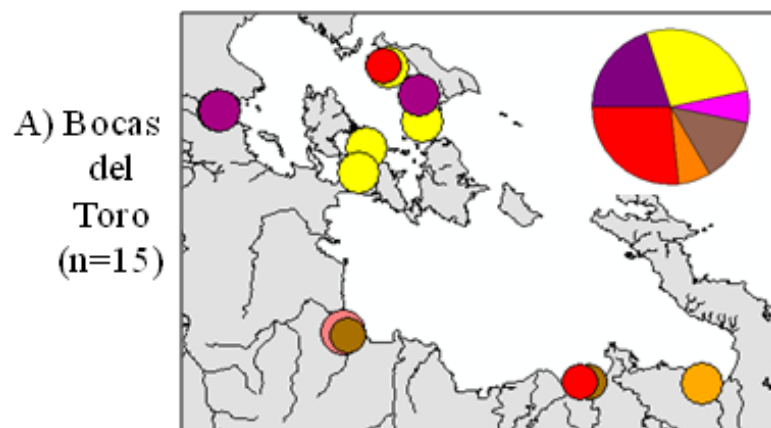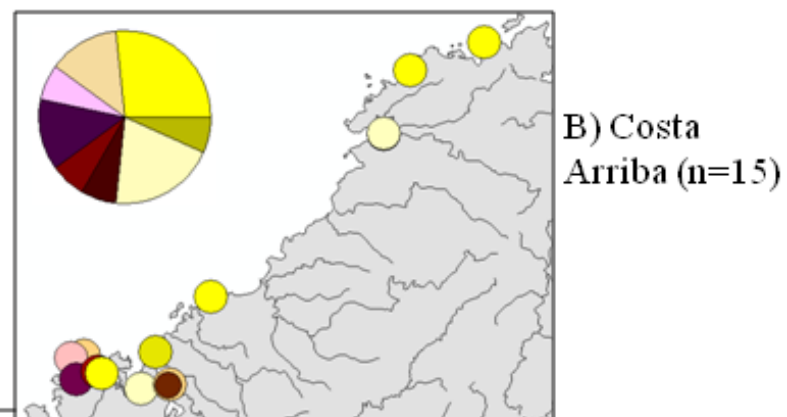

C) Montijo Gulf (n=14)

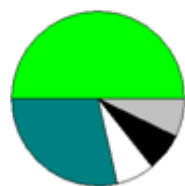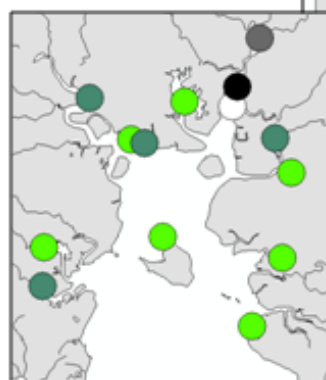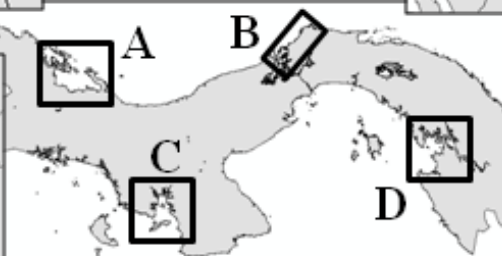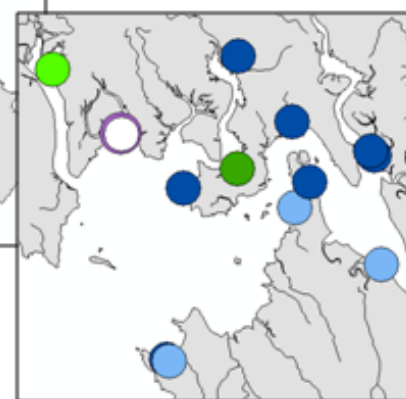

D) San Miguel Gulf (n=14)

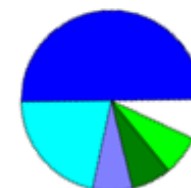

Supplement: Additional file 2 — Median joining network indicating and geographic distribution of cpDNA haplotypes found in Avicennia germinans (Black mangrove). Within the network, the haplotype name and the number of individuals per each haplotype is indicated. In addition, for each estuary, the geographic distribution of haplotypes and their frequency (i.e. pie) is indicated. [file 1471-2148-12-205-S2.pdf]

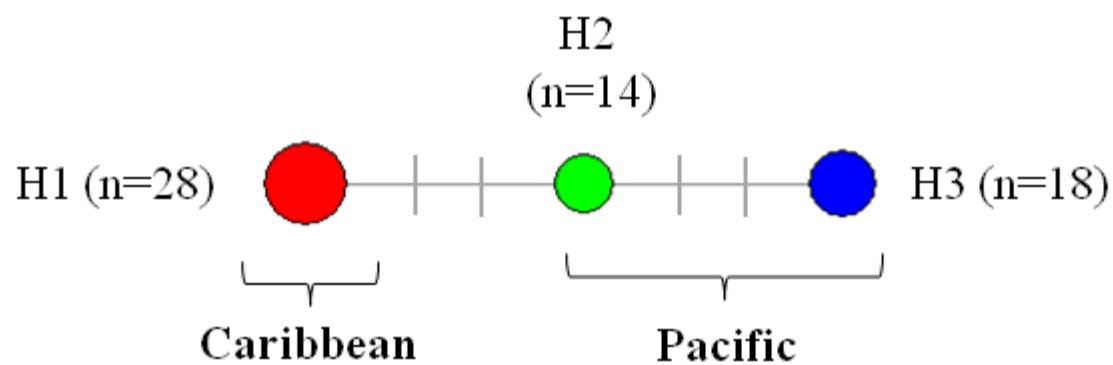

A) Bocas del Toro (n=14)

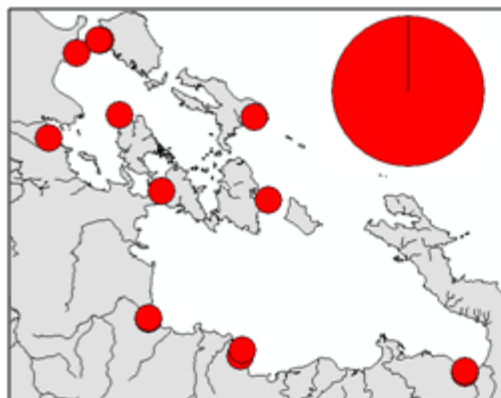

B) Costa Arriba (n=14)

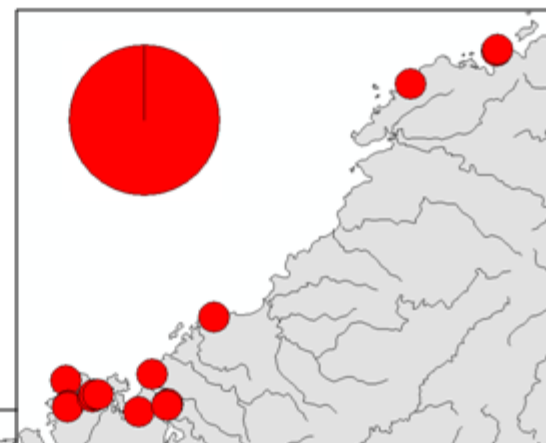

C) Montijo Gulf (n=14)

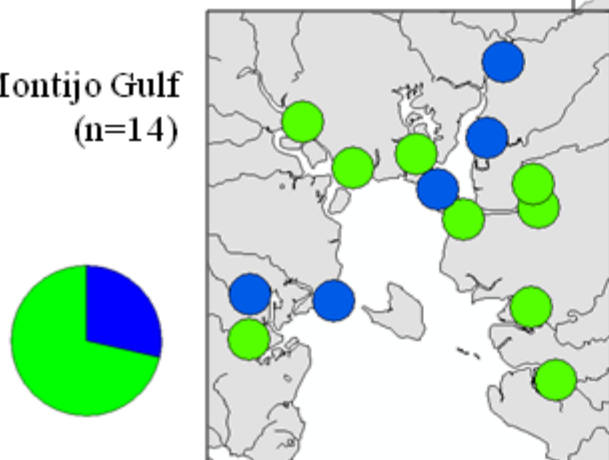

D) San Miguel Gulf (n=18)

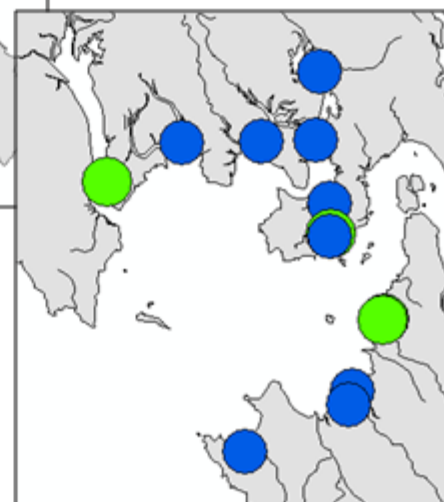

Supplement: Additional file 3 — Median joining network indicating and geographic distribution of cpDNA haplotypes found in Rhizophora mangle (Red mangrove). Within the network the haplotype name and the number of individuals per each haplotype is indicated. In addition, for each estuary the geographic distribution of haplotypes and their frequency (i.e. pie) is indicated. [file 1471-2148-12-205-S3.pdf]
