# Supplementary material for: Comparative genetic structure of two mangrove species in Caribbean and Pacific estuaries of Panama
Source: BMC Evol Biol. 2012 Oct 18;12:205. doi: 10.1186/1471-2148-12-205 (PMC3543234; doi:10.1186/1471-2148-12-205)
Supplement: Additional file 5 — Bayesian genetic assignment of Avicennia germinans (black mangrove) from two Caribbean (Bocas del Toro and Costa Arriba) and two Pacific (Montijo Gulf and San Miguel Gulf) estuaries in Panama based on STRUCTURE ver. 2.2 and GENELAND ver. 2.0.12. The true K for each procedure after simulations is indicated. [file 1471-2148-12-205-S5.pdf]

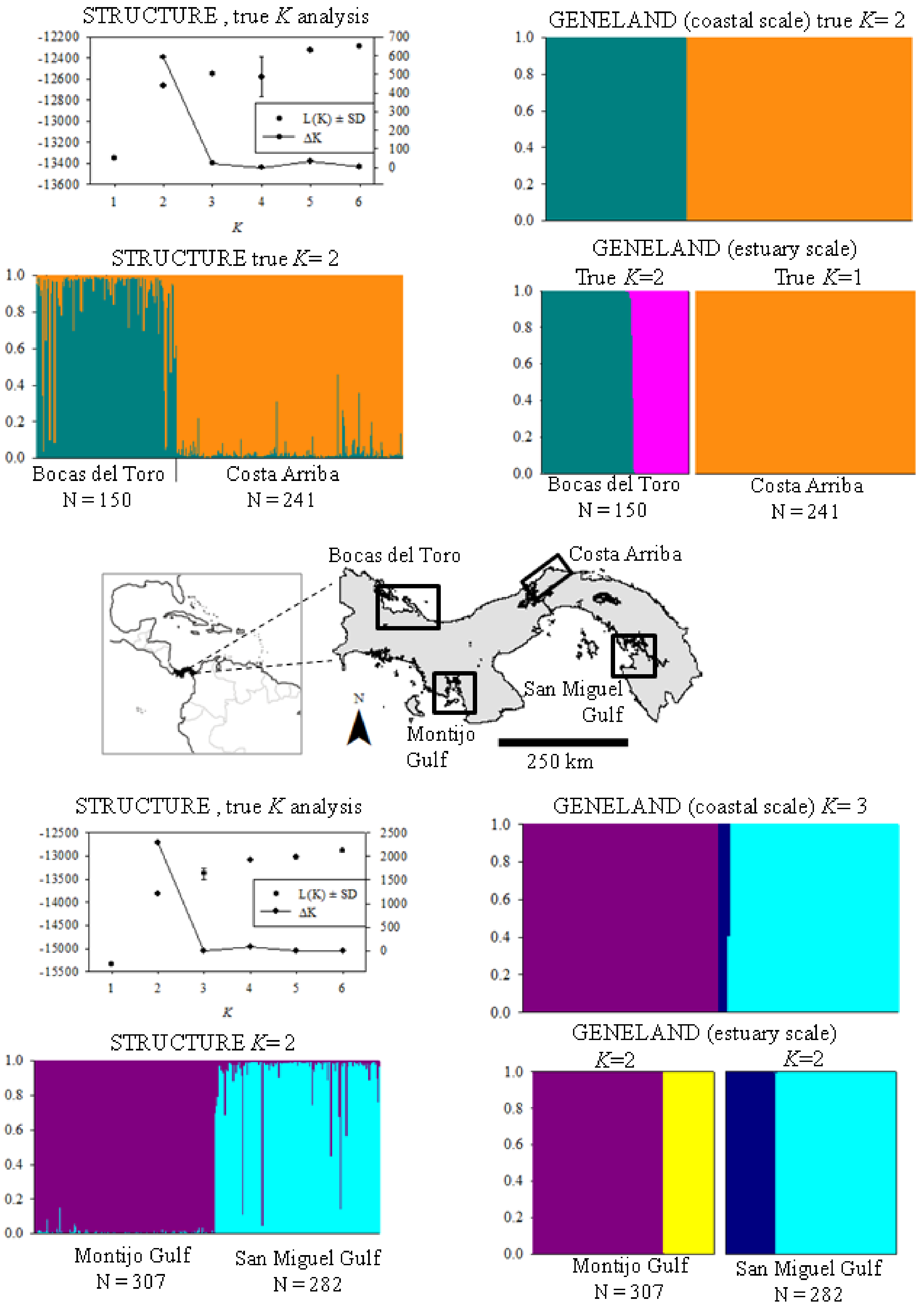
STRUCTURE , true K analysis
L(K) ± SD
ΔK
K
GENELAND (coastal scale) true K= 2
STRUCTURE true K= 2
Bocas del Toro
N = 150
Costa Arriba
N = 241
GENELAND (estuary scale)
True K=2
True K=1
Bocas del Toro
N = 150
Costa Arriba
N = 241
Bocas del Toro
Costa Arriba
San Miguel
Gulf
Montijo
Gulf
N
250 km
STRUCTURE , true K analysis
L(K) ± SD
ΔK
K
GENELAND (coastal scale) K= 3
STRUCTURE K= 2
Montijo Gulf
N = 307
San Miguel Gulf
N = 282
GENELAND (estuary scale)
K=2
K=2
Montijo Gulf
N = 307
San Miguel Gulf
N = 282
