# Supplementary material for: Comparative genetic structure of two mangrove species in Caribbean and Pacific estuaries of Panama
Source: BMC Evol Biol. 2012 Oct 18;12:205. doi: 10.1186/1471-2148-12-205 (PMC3543234; doi:10.1186/1471-2148-12-205)
Supplement: Additional file 6 — Bayesian genetic assignment of Rhizophora mangle (Red mangrove) from two Caribbean (Bocas del Toro and Costa Arriba) and two Pacific (Montijo Gulf and San Miguel Gulf) estuaries in Panama based on STRUCTURE ver. 2.2, INSTRUCT and GENELAND. 2.0.12. The true K for each procedure after simulations is indicated. In addition, INSTRUCT was used to help to simultaneously infer the selfing rates of this mixed mating species and the demic structure on both sides of the Isthmus. [file 1471-2148-12-205-S6.pdf]

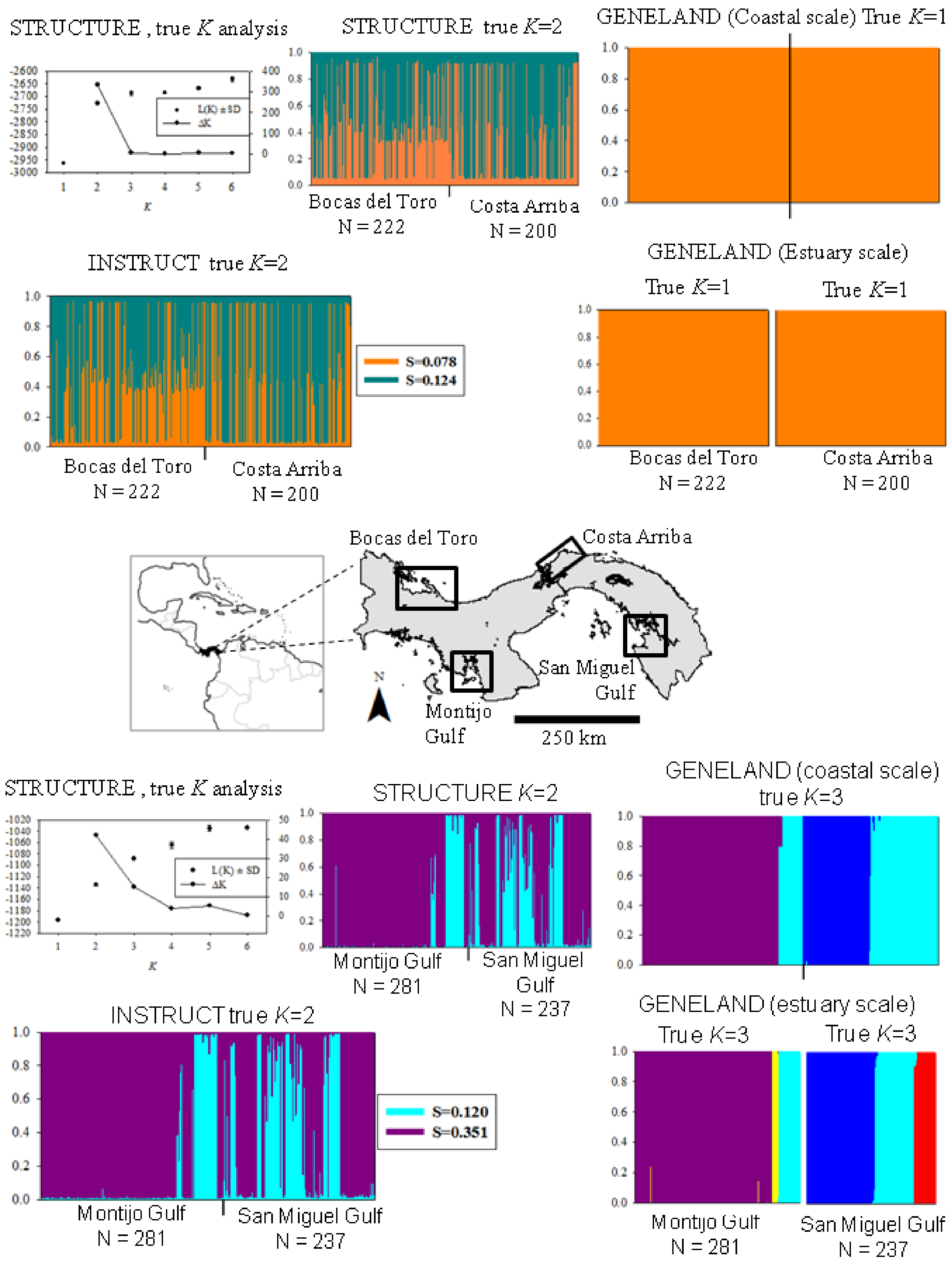
STRUCTURE , true K analysis
L(K) ± SD
ΔK
K
STRUCTURE true K=2
Bocas del Toro
N = 222
Costa Arriba
N = 200
GENELAND (Coastal scale) True K=1
INSTRUCT true K=2
S=0.078
S=0.124
GENELAND (Estuary scale)
True K=1
True K=1
Bocas del Toro
Costa Arriba
N
Montijo Gulf
San Miguel Gulf
250 km
STRUCTURE , true K analysis
STRUCTURE K=2
Montijo Gulf
N = 281
San Miguel Gulf
N = 237
GENELAND (coastal scale) true K=3
INSTRUCT true K=2
S=0.120
S=0.351
GENELAND (estuary scale)
True K=3
True K=3
